# Psychosocial job stressors and risk of suicidal behavior – an observational study among Swedish men

**DOI:** 10.5271/sjweh.4039

**Published:** 2022-08-31

**Authors:** Maria Åberg, Elisabeth Staats, Josefina Robertson, Linus Schiöler, Kjell Torén, Anthony D LaMontagne, Mia Söderberg, Margda Waern, Jenny Nyberg

**Affiliations:** 1 School of Public Health and Community Medicine, Institute of Medicine, Sahlgrenska Academy, University of Gothenburg, Gothenburg, Sweden; 2 Region Västra Götaland, Regionhälsan, Gothenburg, Sweden; 3 Department of Infectious Diseases, Institute of Biomedicine, Sahlgrenska Academy, University of Gothenburg, Gothenburg, Sweden; 4 Region Västra Götaland, Sahlgrenska University Hospital, Gothenburg, Sweden; 5 Occupational and Environmental Medicine, School of Public Health and Community Medicine, Institute of Medicine, The Sahlgrenska Academy, University of Gothenburg, Gothenburg, Sweden; 6 Institute for Health Transformation & School of Health & Social Development, Deakin University, Geelong, Victoria, Australia; 7 Melbourne School of Population and Global Health, The University of Melbourne, Melbourne, Australia; 8 Department of Psychiatry and Neurochemistry, Institute of Neuroscience and Physiology, Sahlgrenska Academy, University of Gothenburg, ­Gothenburg, Sweden; 9 Section for Clinical Neuroscience, Institute of Neuroscience and Physiology, Sahlgrenska Academy, University of Gothenburg, Gothenburg, Sweden

**Keywords:** cohort, epidemiology, JEM, job control, job demand, job exposure matrix, risk assessment, self-harm, suicide, Sweden

## Abstract

**Objective:**

This study aimed to explore the relationship between psychosocial job stressors and suicidal behavior (fatal and non-fatal) among Swedish men while controlling for potential confounders.

**Methods:**

Population-based Swedish longitudinal cohort study of male conscripts without previous self-harm (N=1 483 310) enlisting 1968–2002. Conscription examinations included measures of IQ, stress resilience and psychiatric diagnoses. Job demand–control (JDC) exposure was assessed using the Swedish Job Exposure Matrix linked to specific occupations. Suicidal behavior among men aged 30–64 was identified in the National Hospital Register (non-fatal self-harm) and Swedish Cause of Death Register (suicide) during follow-up 2002–2014. Cox regression models were used to estimate associations between JDC category and suicidal behavior.

**Results:**

In fully adjusted models, passive jobs (low demand-low control) showed the highest risk of suicidal behavior [hazard ratio (HR) 1.33, 95% confidence interval (CI) 1.25–1.43] compared to those with low strain (low demand-high control), followed by high strain (high demand-low control) (HR 1.12, 95% Cl 1.03–1.22). A lower risk of suicidal behavior was found in the active category, where levels of both demand and control are high (HR 0.64, 95% Cl 0.60–0.70). Separate analyses for suicide as outcome revealed a lower risk of suicide in persons with active jobs (high demands-high control). The passive category showed a higher risk for suicide, but the association did not remain after adjustment for stress resilience and IQ.

**Conclusions:**

These results show that psychosocial job stressors among men are associated with risk for suicidal behavior. Improving job control has the potential to decrease suicidal behavior for this group.

A pattern of male predominance in death by suicide is seen in most western countries (1). Men tend to under-report mental ill-health, more seldom seek professional help, and also use more lethal suicide methods than women (2). In the US, suicide rates have been rising over the last 15 years among men of all ages, and this phenomenon is most pronounced in midlife (3). In Sweden, the suicide rate for midlife men (26.5 per 100 000 in 2020; National Board of Health and Welfare, 2021) is on par with that observed for older men (≥65 years). Improved understanding of the modifiable determinants of suicide in this group could inform preventive interventions.

In recent years increasing evidence shows that job stressors affect mental health (4, 5). High level of chronobiological stress (overtime, night or shift work) or adverse physical working conditions (physically challenging or dangerous work), are considered possible contributing factors to suicide (6). Research on how psychosocial job stressors affect suicidal behavior is scarce in general and among men of working age in particular (6–9).

A broadly used theoretical model for understanding psychosocial job stressors in relation to stress and health is the job demand–control (JDC) model (10). Job demand alludes to work intensity and time pressure, while job control refers to how much freedom a person has to control and organize their own work. Jobs that entail high demands in combination with low control are known as high strain jobs, which are associated with adverse health outcomes including cardiovascular disease and depression in men (11, 12). One approach to the measurement of psychosocial job stressors is the use of a job exposure matrix (JEM) (13). This approach surveys individuals within different occupations and aggregates their responses regarding exposures on an occupational level. Each occupation can then be assigned an index score and these scores can be used in population-level studies in combination with linked health outcome data.

Most studies investigating the effect of psychosocial job stressors on suicidal behavior are cross-sectional and observational in design, and there is a need for longitudinal research (14). A life course perspective is important as phenomena in earlier life may confound the relation between job stressors and suicidal behavior (15–17). For example, this can occur through confounding by selection, in which early life experience of mental disorder and/or traumatic life events can result in lower educational attainment and thus employment in lower skilled poorer quality jobs. In this scenario, early life experience could be a common prior cause of both exposure and outcome.

A previous study suggests that individuals who make suicide attempts and those who die by suicide should be considered as two distinct populations that partially overlap. Compared to suicide attempters, those who died by suicide were more likely to be males living alone. They were also more likely to have been followed by a primary care provider, more frequently presented somatic problems, and made use of more violent methods (18). Suicidal behavior in working-age may also differ from those of younger adults. The latter group has a higher risk of suicide attempts, while the risk of suicide is greater with increasing age. Risk profiles for fatal and non-fatal outcomes such as self-harm may overlap more in working age than among young adults, paralleling results reported for elderly individuals (19). Therefore, it is important to also separately analyze suicide mortality. There are few prospective data cohorts large enough to yield adequately-sized samples of individuals who died by suicide to detect effects of risk factors over the life course. To address this need, the current study utilized a longitudinal design involving a large national cohort of late-adolescent enlisting men with no history of self-harm. Based on Karasek’s JDC model (10), we hypothesize that high job demands and low control will relate to an increased risk of suicidal behavior. We expect these associations to be partially explained by potential confounders such as parental education (20), cognitive performance (21, 22) and stress resilience (23). Also, mental illness during adolescence may interfere with both choices of occupation and later suicidal behavior which should be taken into account (24).

## Methods

### Psychosocial job exposure

A JEM (13, 25) was used to estimate psychosocial job stressors for each occupation. The JEM was developed in 2000 using questionnaire data from the Swedish Work Environment Survey 1989–97 assessing psychosocial job conditions in randomly selected participants aged 16–64 years (N=48 894). Items measuring job control and demand were identified by factor analyses in which occupational code, sex, age and duration of work were used to construct the matrix. The JEM assigns separate estimates of demand and control for 320 occupations by age group (30–44 years and 45–64 years in the present study).

Job demands were measured with five items; decision latitude (control) with six (25). All items were scored using a scale (1–10). The JDC category was updated each year by occupational information from the Longitudinal Integration Database for Health Insurance and Labor Market Studies (Swedish acronym LISA). The scores were then dichotomized into high and low, using the median of the distribution as cut-off. Combining demand and control with the median cut-offs divided the participants into four categorical quadrants; high strain (high demand-low control), active (high demand-high control), passive (low demand-low control) and low-strain (low demand-high control).

### Design and exposure

We performed a population-based prospective study of late-adolescent men who underwent compulsory military conscription examinations between 1968 and 2002. At enlistment, all men underwent an examination at one of six regional conscription centers. The examinations included assessment of physical health status and medical examination by a physician, as well as a mental health assessment by a board-certified psychologist. Individual register data from several Swedish national registers were linked through the unique personal identification number assigned to each registered person in Sweden.

Occupation was classified in occupational codes each year during 2001–2013 using from the LISA database, which is updated annually and includes data for all Swedish residents aged ≥16 years.

The exposure – work demand and control – was assessed using JEM based on yearly updated occupations during 2001–2013 (13). The follow-up period covered ages 30–64 (2002–2014), an age group defined as working age (3). Study outcomes were suicidal behavior (fatal and non-fatal self-harm) and suicide only. Possible confounding effects of conscription age and year, parental education, stress resilience and cognitive function were also investigated. After linkage, all data were anonymized, and individuals were assigned a unique identifier by Statistics Sweden in order to maintain confidentiality. The authors assert that all procedures contributing to this work comply with the ethical standards of the relevant national and institutional committees on human experimentation and with the Helsinki Declaration of 1975, as revised in 2008. All procedures involving human subjects were approved by the Ethics Committee of the University of Gothenburg (462-14) and Confidentiality Clearance at Statistics Sweden. All authors have followed the ICMJE (International Committee of Medical Journal Editors) guidelines.

### Study population

**Figure 1:**
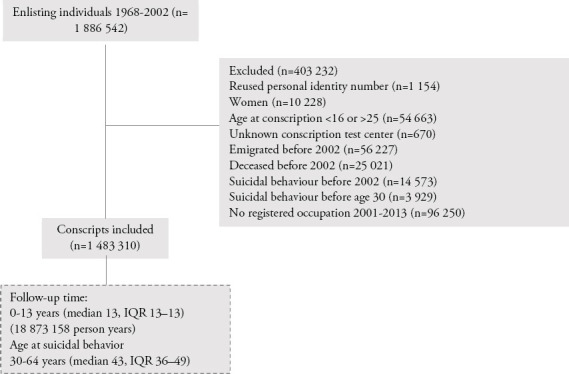
The figure shows the flow chart of included and excluded conscripts, median years of observation (follow-up time) and age at suicidal behavior with interquartile ranges (IQR) and total person years of observation.

The source population comprised all conscripts (N=1 886 542) born 1950–1984. Only individuals with severe chronic medical or mental conditions, serious disabilities or incarcerated individuals were granted exemption (in all, 2–3% of the male population per year). Male conscripts aged 16–25 years old at conscription, with a mean age of 18.2 [standard deviation (SD) 0.7] years, were included in the study. To reduce the risk of possible reverse causation, men with registered episodes of non-fatal self-harm [diagnostic codes E950-E959 (ICD-8/9) and X60-X84 (ICD-10)] prior to 2002 or age 30 were excluded from the analyses (N=18 502, figure 1). Additional exclusions were made for men who died or emigrated before 2002, men with reused personal identity number, unknown conscription test center or no registered occupation during 2001–2013, yielding a total of 1 483 310 men (figure 1).

### Covariates

*Conscription year*. There might be effects of variation in diagnosis frequency and differences in conscription procedures depending on what year the subject enlisted. For example, as the psychiatric indications for exemption from conscription examination broadened over time, the proportion with common mental disorders decreased over the study period from 9.1% (-1970) to 0.7% (2001). Also, the IQ tests (see below) were slightly amended in 1980. Therefore, conscription year was obtained from the Swedish Military Service Conscription Register and included as a covariate to the analyses.

*Parental education*. Socioeconomic status early in life may affect both choice of occupation and risk of suicidal behavior (26, 27). As socioeconomic predictor we adjusted the analyses for highest achieved parental education identified using the annually updated LISA database. Maternal and paternal education were graded separately in 3 levels: pre-high school education (up to 9 years), high school education and university/postgraduate education.

*Stress resilience*. Stress resilience was assessed at enlistment during a 25–30-minute semi-structured interview performed by a psychologist. Inter-rater reliability was high (r =0.86) (28, 29). The original objective of the interview was to estimate the conscript’s ability to cope with the psychological requirements of military service and war-time stress, based on how they had dealt with past life events (28, 29). The questions focused on the conscript’s civilian environment and covered school and work experience, leisure activities, home environment, upbringing and emotional stability. For example, willingness to assume responsibility, independence, extraversion/introversion, persistence, emotional stability, power of initiative, ability to adjust, social skills, adjustment problems, conflicts and social maturity were assessed. Instead of measuring a specific trait, this measure aims to capture a specific function, ie, the ability to function in a demanding and stressful environment. Characteristics that gave a low stress resilience score include neurotic tendencies, antisocial personality, adjustment problems, introversion, undemocratic values, obsessive interest in the military and violent or aggressive behavior (28). This measure has been suggested to capture a stress resilience dimension similar to that rated with the Connor-Davidson Resilience Scale (CD-RISC) and the Resilience Scale for Adults (30). It has also been argued that a high stress resilience rating corresponds to low neuroticism, high conscientiousness, and high extraversion, thus similar to the ‘general factor of personality’ (31). Stress resilience was rated and assigned a summary stanine (or ‘standard nine’) score of 1–9. The stanine scores were then categorized as low (score 1–3), medium (score 4–6) and high (score 7–9). The interview has been described more in detail (28, 31, 32), but the actual test manuals remain classified military material.

### Cognitive performance (IQ)

At enlistment, subtests of four different cognitive domains were performed: verbal, visuospatial, logical-inductive and technical cognition. The cognitive performance tests are described in detail elsewhere (17). Non-weighted results from the subtests were combined to create a general measure of cognitive performance. All test results were standardized against data from previous years, resulting in stanine scores 1–9. We categorized them as low (score 1–3), medium (score 4–6) and high (score 7–9).

### Psychiatric diagnoses

If the psychologist noted psychiatric symptoms during the conscription interview, the conscript was further evaluated by the conscription physician, and psychiatric disorders were diagnosed according to the International Classification of Diseases (ICD). For the current study, men with psychiatric disorders at conscription were also identified through the Swedish National Hospital Register, which contains both inpatient and outpatient diagnoses recorded in a hospital setting, including referrals to specialists and emergency visits. In Sweden, it is mandatory for all private and publicly funded hospitals to register one principal discharge diagnosis and up to thirty contributory diagnoses. Register coverage for all inpatient care increased gradually during 1968–1986 and diagnoses from hospital-based outpatient care have been recorded since 2001. The ICD diagnoses validated (N=132, including several psychiatric diagnoses) had positive predictive values for correct diagnoses of 85–95% (33).

### Outcomes

Two separate outcomes were studied: (i) suicidal behavior, a composite variable of first registered fatal or non-fatal self-harm event and (ii) death by suicide. Suicidal behavior and suicides occurring at ages 30–64 (ie, 1 January 2002 to 31 December 2014) were thus included in separate models. Suicides (ICD-10 X60-X84) were identified through the Swedish Cause of Death Register which is maintained at the National Board of Health and Welfare. This register covers all deaths since 1961 and is annually updated based on death certificate diagnoses. First obtained ICD-code for non-fatal self-harm (X60-X84) was accessed from the National Hospital Register, described above. The register does not distinguish between suicide attempts and non-suicidal self-injury, and our outcome hence encompasses both.

### Statistical analysis

Cox proportional hazard models were used to estimate the hazards ratios (HR), and 95% CI for suicidal behavior in working aged men by JDC category (reference: low strain ie, low demand-high control). Age was used as time scale in all models. The JDC exposure was treated as a time dependent, or time-varying, variable, updated yearly with the value from the prior year. In addition to unadjusted model I, conscription calendar year and parental education were included as potential confounders in model II. Model III was additionally adjusted for stress resilience and model IV for cognitive performance [intelligence quotient (IQ)]. We also constructed models excluding conscripts with a psychiatric diagnosis (ICD-10; F00-F99) at time of conscription. Analyses were repeated using death by suicide or non-fatal suicidal behavior as outcomes. Also separates analyses were performed with the job strain components (low job control / low job demand) and risk of suicidal behavior (reference: high control / high demand respectively). The proportional hazard assumptions were investigated using tests and plots based on weighted residuals. JDC categories active and passive, parental education, IQ and stress resilience showed signs of non-proportional hazards; but further investigation showed a negligible effect on the estimates. Therefore, JDC categories parental education, IQ and stress resilience was used in the models without any remedial measures.

The follow-up period began in 2002 and person-time was included until time of outcome (first episode of non-fatal self-harm or death by suicide), death by other causes, emigration, age 64 or end of follow-up on 31 December 2014, whichever happened first. A P-value of <0.05 was considered statistically significant. All statistical calculations were performed with SAS version 9.4 (SAS Institute, Cary, NC, USA).

## Results

Psychological characteristics and parental education at conscription are shown by JDC category in table 1. Men in occupations associated with low demand-low control (ie, the passive JDC category) had lower parental education, stress resilience and IQ compared to all others. They also had higher frequencies of psychiatric disorders compared to the active and low strain JDC category.

**Table 1:** Characteristics at conscription by job demand–control (JDC) category among 1 483 310 male conscripts. [SD=standard deviation.]

	All		Active		High strain		Low strain		Passive	
				
	N (%)	Mean (SD)	N (%)	Mean (SD)	N (%)	Mean (SD)	N (%)	Mean (SD)	N (%)	Mean (SD)
Men	1 483 310		480 120 (32.4)		196 534 (13.3)		228 837 (15.4)		577 819 (39.0)	
Age (years)		18.3 (0.7)		18.3 (0.6)		18.3 (0.7)		18.3 (0.7)		18.3 (0.7)
Parental education										
Low	416 901 (28.8)		105 081 (22.4)		54 504 (28.5)		68 580 (30.7)		188 736 (33.5)	
Medium	634 928 (43.9)		190 331 (40.5)		80 696 (42.2)		101 998 (45.7)		261 903 (46.5)	
High	395 725 (27.3)		174 319 (37.1)		55 852 (29.2)		52 751 (23.6)		112 803 (20.0)	
Stress resilience										
Low	251 867 (18.4)		46 654 (10.2)		35 428 (19.6)		38 859 (18.5)		130 926 (25.3)	
Medium	816 847 (59.8)		266 330 (58.3)		106 525 (58.9)		129 349 (61.4)		314 643 (60.7)	
High	298 260 (21.8)		144 121 (31.5)		38 969 (21.5)		42 308 (20.1)		72 862 (14.1)	
IQ										
Low	292 713 (20.5)		33 160 (7.1)		40378 (21.3)		46 654 (21.1)		172 521 (31.3)	
Medium	784 966(54.8)		239 267 (51.0)		104 844 (55.3)		132 453 (60.0)		308 402 (55.9)	
High	353 450 (24.7)		196 740 (41.9)		44 456 (23.4)		41 585 (18.8)		70 669 (12.8)	
Psychiatric diagnosis at conscription	57 888 (3.9)		13 253 (2.8)		9013 (4.6)		9357 (4.1)		26 265 (4.5)	

During follow-up, 2335 men aged 30–64 died by suicide. At least one self-harm event was recorded in the National Hospital Register for 7334 men.

HR for suicidal behavior (fatal or non-fatal) by JDC category are shown in table 2. Passive work was related to the highest risk (HR 1.33, 95% Cl 1.25–1.43) compared to the low strain category, followed by the high strain category (high demand-low control) (HR 1.12, 95% Cl 1.03–1.22) in fully adjusted models. A lower risk of suicidal behavior was found in the active group, where levels of both demand and control are high (HR 0.64, 95% Cl 0.60–0.70). Separate analyses excluding all individuals with a psychiatric diagnosis at time of conscription showed similar results (table 3). Separate analyses for non-fatal suicidal behavior (ie, suicide attempts) showed increased risk in the passive and high strain quadrants and decreased risk in the active quadrant compared to the low strain category (supplementary material, www.sjweh.fi/article/4039, tables S1 and S2).

**Table 2:** Hazard ratios (HR) for suicidal behavior (fatal and non-fatal) among working aged men, by job demand–control (JDC) category. For each exposure category number of events during follow-up (No events) and number of events per 10 000 person-years are included. [CI=confidence interval.]

	Model I ^a^		Model II ^b^		Model III ^c^		Model IV ^d^	
			
	HR (95% CI)	Events (N)/ per 10 000 person-years	HR (95% CI)	Events (N)/ per 10 000 person-years	HR (95% CI)	Events (N)/ per 10 000 person-years	HR (95% CI)	Events (N)/ per 10 000 person-years
Events/population		8431/1 476 341		8162/1 440 753		7419/1 328 058		7374/1 322 425
JDC category								
Active	0.51 (0.47–0.55)	1683/3.0	0.54 (0.50–0.58)	1632/3.0	0.60 (0.56–0.65)	1533/2.9	0.64 (0.60-0.70)	1531/2.9
High strain	1.07 (0.99–1.15)	1242/5.9	1.11 (1.03–1.21)	1205/5.9	1.12 (1.03–1.22)	1112/5.9	1.12 (1.03-1.22)	1105/5.9
Passive	1.49 (1.40–1.58)	4227/7.8	1.48 (1.38–1.57)	4100/7.8	1.37 (1.28–1.47)	3658/7.7	1.33 (1.25-1.43)	3628/7.7
Low strain	1 (reference)	1279/5.5	1 (reference)	1225/5.4	1 (reference)	1116/5.3	1 (reference)	1110/5.3

a Unadjusted.

b Adjusted for conscription year and parental education.

c Model II + additionally adjusted for stress resilience.

d Model III+ additionally adjusted for IQ

**Table 3:** Hazard ratios (HR) for suicidal behavior (fatal and non-fatal) among working aged men, by job demand–control (JDC) category excluding men with a psychiatric diagnosis (F-diagnosis) at time of conscription. [CI=confidence interval.]

	Model I ^a^		Model II ^b^		Model III ^c^		Model IV ^d^	
			
	HR (95% CI)	Events (N)/ per 10 000 person-years	HR (95% CI)	Events (N)/ per 10 000 person-years	HR (95% CI)	Events (N)/ per 10 000 person-years	HR (95% CI)	Events (N)/ per 10 000 person-years
Events/population		7735/1 418 703		7497/1 386 124		6846/1 279 838		6806/1 274 504
JDC category								
Active	0.51 (0.47–0.55)	1591/2.9	0.54 (0.50–0.59)	1544/2.9	0.60 (0.55–0.65)	1459/2.9	0.64 (0.59-0.69)	1457/2.9
High strain	1.06 (0.98–1.15)	1137/5.7	1.11 (1.02–1.20)	1104/5.6	1.11 (1.02–1.21)	1024/5.7	1.12 (1.02-1.22)	1017/5.7
Passive	1.47 (1.38–1.57)	3827/7.4	1.46 (1.37–1.56)	3718/7.4	1.36 (1.26–1.45)	3324/7.3	1.32 (1.23-1.41)	3298/7.3
Low strain	1 (reference)	1180/5.3	1 (reference)	1131/5.2	1 (reference)	1039/5.1	1 (reference)	1034/5.1

a Unadjusted.

b Adjusted for conscription year and parental education.

c Model II + additionally adjusted for stress resilience.

d Model III+ additionally adjusted for IQ

Results from the fully adjusted analyses for the suicide outcome revealed a lower risk of suicide in the active category (HR 0.59, 95% CI 0.52–0.68) compared to low strain (table 4). Persons with passive jobs showed a higher risk compared to the reference category but this association was not significant when adjusted for stress resilience and IQ. Separate analyses excluding all individuals with a psychiatric diagnosis at time of conscription showed similar results (table 5).

**Table 4:** Hazard ratios (HR) for suicide among working aged men, by job demand–control (JDC) category. [CI=confidence interval.]

	Model I ^a^		Model II ^b^		Model III ^c^		Model IV ^d^	
			
	HR (95% CI)	Events (N)/ per 10 000 person-years	HR (95% CI)	Events (N)/ per 10 000 person-years	HR (95% CI)	Events (N)/ per 10 000 person-years	HR (95% CI)	Events (N)/ per 10 000 person-years
Events/population		2283/1 477 347		2200/1 441 720		2071/1 328 941		2060/1 323 303
JDC category								
Active	0.53 (0.46-0.60)	537/1.0	0.54 (0.48–0.62)	520/0.95	0.58 (0.50–0.66)	502/0.96	0.59 (0.52–0.68)	501/0.96
High strain	0.95 (0.82-1.10)	342/1.6	0.99 (0.86–1.15)	336/1.6	0.99 (0.85–1.15)	319/1.7	1.00 (0.85–1.16)	317/1.7
Passive	1.17 (1.04–1.31)	1006/1.9	1.17 (1.04–1.31)	965/1.8	1.11 (0.98–1.26)	891/1.9	1.10 (0.97–1.24)	885/1.9
Low strain	1 (reference)	398/1.7	1 (reference)	379/1.7	1 (reference)	359/1.7	1 (reference)	357/1.7

a Unadjusted.

b Adjusted for conscription year and parental education.

c Model II + additionally adjusted for stress resilience.

d Model III+ additionally adjusted for IQ.

**Table 5:** Hazard ratios (HR) for suicide among working aged men, by job demand–control (JDC) category excluding conscripts with a psychiatric diagnosis (F-diagnosis) at time of conscription. [CI=confidence interval.]

	Model I ^a^		Model II ^b^		Model III ^c^		Model IV ^d^	
			
	HR (95% CI)	Events (N)/ per 10 000 person-years	HR (95% CI)	Events (N)/ per 10 000 person-years	HR (95% CI)	Events (N)/ per 10 000 person-years	HR (95% CI)	Events (N)/ per 10 000 person-years
Events/population		2143/1 419 597		2070/1 386 982		1957/1 280 631		1947/1 275 293
JDC category								
Active	0.52 (0.46–0.60)	514/0.94	0.54 (0.47–0.62)	498/0.93	0.57 (0.49–0.66)	482/0.95	0.59 (0.51–0.68)	481/0.95
High strain	0.95 (0.82–1.10)	319/1.6	0.98 (0.84–1.14)	313/1.6	0.99 (0.85–1.15	300/1.7	0.99 (0.85–1.16)	298/1.7
Passive	1.16 (1.03–1.31)	935/1.8	1.17 (1.03–1.32)	901/1.8	1.11 (0.98–1.26)	835/1.8	1.10 (0.97–1.25)	830/1.8
Low strain	1 (reference)	375/1.7	1 (reference)	358/1.6	1 (reference)	340/1.7	1 (reference)	338/1.7

a Unadjusted.

b Adjusted for conscription year and parental education.

c Model II + additionally adjusted for stress resilience.

d Model III+ additionally adjusted for IQ.

**Figure 2:**
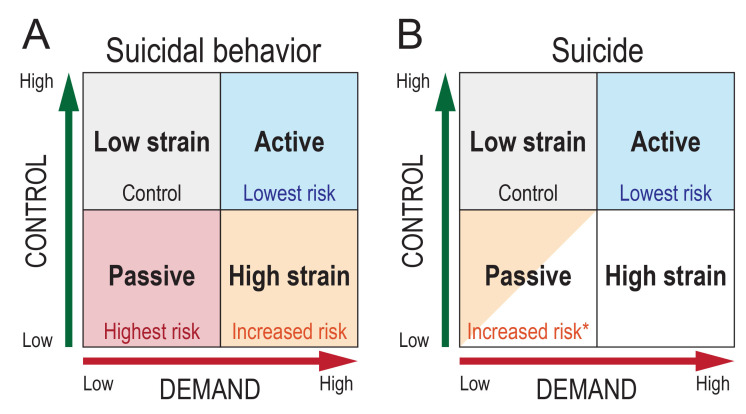
Summary of the results. * Not significant when adjusted for stress resilience and IQ.

The results from analyses including men with psychiatric diagnoses at time of conscription are summarized in figure 2. Increased risk in the high strain and the passive quadrant and decreased risk in the active quadrant indicate that it is mainly low job control that is associated with risk of suicidal behavior, whereas high job demands might be associated with a lower risk of suicidal behavior. Analyses on the job strain components separately confirmed this (supplementary tables S3–4).

## Discussion

### Main findings

In this register-based study of approximately 1.5 million Swedish men, we found that passive occupations, that is, those with low job demands and low job control, were associated with an increased risk of suicidal behavior compared to the low strain category (low demand-high control). Also, the high strain category (high demand-low control) was associated with an increased risk of suicidal behavior compared to the low strain category. These associations remained even after adjusting for a number of potential confounders including parental education, stress resilience and IQ as well as excluding men with adolescent psychiatric disorders. Having an active job, defined as high levels of both demand and control, was associated with a decrease in the risk of any suicidal behavior, as well as decreased risk of suicide. The passive category was associated with elevated risk of suicide, but the association did not remain after adjustment for stress resilience and IQ.

### Comparison with findings from other studies

To our knowledge, this is the first study presenting longitudinal data of a job demand control model and fatal and non-fatal suicidal behavior using the composite outcome for comparison. One longitudinal questionnaire study comparing fatal and non-fatal suicide attempts separately in western Canadian sawmill workers (N =28 794) showed that low demand was associated with increased odds for both suicide and attempted suicide (8). However, the association did not remain for attempted suicide after controlling for sociodemographic and non-psychosocial job stressor variables. In the present study, associations between passive jobs and suicidal behavior were only slightly attenuated when adjusting for potential confounders, suggesting that low job control and low job demands constitute independent modifiable risk factors.

Our separate analyses of the suicide outcome, revealed a lower risk of suicide in the active category. The passive category showed a higher risk compared to the reference category but was not significant when adjusted for stress resilience and IQ. There are a few longitudinal studies of associations between JDC and suicide for comparison. Our findings contrast with results from a German survey study reporting no associations between job strain and suicide during the 13 years of follow-up (6). However, low statistical power (28 suicide cases) and pooled analyses of men and women might explain part of the difference. The current results are consistent with a recent large longitudinal study from France showing that, for men, passive jobs and job strain were significant risk factors for suicide over 26 years of follow-up (9). Our results are also in line with a large case–control study from Australia that assessed the relationship between psychosocial job stressors and suicide mortality across the national working population (34). Among 9010 cases and 14 007 matched controls, the results suggest that low job control was associated with increased odds of male suicide after adjusting for socioeconomic status. Also, in a multicenter community-based Japanese cohort study that employed a JDC model questionnaire, low control at work was revealed as a predictor of suicide among male workers (7).

High strain jobs (high demand-low control) were associated with a moderate increase in risk of suicidal behavior in our male cohort. However, higher job demands in active jobs were not associated with an increased risk. Moreover, separate analyses on the job strain components confirmed that high job demand per se was associated with a decrease in the risk of suicidal behavior. This contrasts to the Australian case–control study (34) where high job demands were associated with increased odds of male suicide after adjusting for socioeconomic status. The longitudinal French study reported a lack of association between job demand and risk for suicide among men (9). This was the case in the Japanese cohort as well, but the number of suicides was very limited (7). Our findings parallel the study of the western Canadian sawmill workers where low demand was associated with increased odds for suicide (8). Possible explanations for these discrepancies could be differences in study design, how suicide attempts and suicides are identified, disparities between countries in labor market policies, working culture, and secular trends. For example, in the modern Swedish labor market, higher demands are often linked to high-status jobs, which may explain the lower risk estimates (35).

### Possible mechanisms for the observed associations

The patterning of exposures by potential confounders revealed higher levels of parental education, stress resilience and IQ in persons with active jobs. Passive jobs showed the opposite pattern. The present data revealed associations with passive jobs and increased risk for suicide, but the risk was attenuated and did not reach significance when adjusting for adolescent stress resilience and IQ. We consider that this is a key contribution of the present study. We have previously shown that psychological variables in young adulthood including low stress resilience (also when adjusting for IQ) and mental illness are associated with the risk for suicide in working aged men (23). Moreover, a recent study suggested that adolescent-onset mental illness was associated with poorer employment outcomes, significantly increased risk of employment in low-skilled occupations, as well as reduced monthly wage earnings (16). It has also been shown that adolescent experience of mental illness may predict worse psychosocial employment conditions in adulthood (36). Thus, we might speculate that adolescent psychological health could confound the associations between psychosocial job stressor and suicidal behavior and that young people with mental health vulnerabilities such as low stress resilience and previous mental illness might be more apt to work in adverse psychosocial environments, which further increases their risk for suicide.

That higher job demands showed moderate (high strain jobs) or no (active jobs) associations with suicidal behavior partially deviates from our hypothesis based on the theoretical model of job strain (10). It may be that jobs classified as having high demands in our study benefit individuals through challenges and opportunities, and thus may be related to a decreased risk of suicidal behavior. The present study examines only men, and men and women may react differently regarding high demands. Women are more likely to report high-strain jobs (37) and have been found to have lower job control compared to men (38). Perhaps high demands among men more strongly correlates with high control. A recent study also using the Swedish JEM showed that higher job demands slightly decreased the risk for depression in men (12), a recognized risk factor for suicidal behavior in working aged individuals. Whether mental illness including depression lies in the trajectory between psychosocial job stressors and suicidal behavior remains to be investigated. Problematic workplace experiences may lead to perceived burdensomeness and feelings of thwarted belongingness, which could contribute to the development of suicidal behavior in vulnerable persons, in accordance with the Interpersonal Theory of Suicide (39). Taken together, the present data show that psychosocial job stressors in men are associated with risk for both fatal and non-fatal suicidal behavior. Men with passive jobs are at increased risk for suicidal behavior compared to men with low strain jobs, whereas active jobs are associated with reduced risk.

### Strengths and limitations

The present study expands on previous findings into a Swedish setting with a large sample size, a prospective and population-based design, data on both suicide attempts and fatal outcomes and also including late adolescent risk factors. The current study has a long follow-up time and uses national, high quality registers with enough power to identify working age men with fatal and non-fatal suicidal behavior. Another strength is the reliance on psychologists and physicians for assessment of adolescent suicidal history, allowing us to exclude individuals with prior self-harm, thereby reducing the risk of reverse causation.

One limitation of our research approach is that we lack individual level work environment data. JEM scores are derived from aggregated data, and interindividual variation in exposure levels within particular occupations cannot be taken into consideration. Thus, it is difficult to elaborate if job exposure or working in a certain occupation is the main determinant. The measurements provided by a JEM will lack precision compared to individual level accounts but are associated with lower likelihood of bias, as they are more likely to represent the “average” experience of job stress across a given occupation (34). Also, stressors considered in the job demand-control model do not cover the whole spectrum of work-related psychosocial factors that might represent risk factors for suicidal behavior (eg, factors considered in the effort-reward imbalance or aspects such as job insecurity and organizational change). A recent Swedish study suggests that job insecurity is associated with an increased risk of suicide (40).

Another limitation is the assumption that questionnaire data from the Swedish Work Environment Survey data collected in 1989–1997 reflects conditions and occupational distribution during the period of observation 2001–2013. However, since occupation data was collected each year during 2001–2013, we were able to assign time-varying exposures over the period of observation.

As always in observational studies, the possibility of residual confounding by unmeasured factors cannot be excluded and our results should be interpreted with caution. For example lack of physical activity during mid-life has been shown to be associated with suicidal behavior (41) and a recent study from Sweden revealed cardiovascular fitness differences in different occupational groups (42).

Incidence of self-harm may be underestimated due to the use of the hospital register, which lacks data from primary care or from individuals not seeking care. Our term “self-harm” includes deliberate self-harm both with and without suicidal intent, since the hospital register does not make a distinction between these two. As non-suicidal self-harm is less prevalent in midlife compared to young adulthood (19), we do not anticipate that this is a major issue.

Although it was mandatory for all men by law to perform the conscription tests, 2–3% of the male population did not conscribe due to imprisonment or severe chronic somatic or mental conditions or functional disabilities (43). This might produce a selection bias favoring subjects without severe illness to the extent that these individuals participate in paid employment.

The current cohort is homogenous regarding gender and ethnicity which limits its generalizability for women and for working men in other populations.

### Implications for policy and practice

Suicide in working age is a significant health concern. The current study offers important information regarding occupational psychosocial risk factors for suicidal behavior in working aged men, emphasizing the need for clinicians to account for pre-employment experiences as well as for long-term job stressors when assessing potential suicide risk. The present findings may stimulate future interventions aiming at improving job control or finding other ways to support those with passive jobs, which could potentially decrease premature death by suicide among working men. Preventive actions targeting this group are of particular importance because men often do not seek help for mental ill-health. A synthesis of systematic reviews stated that the effects of interventions that increase job control are usually positive for the organization in terms of absenteeism, financial benefit and productivity or performance (44). Interventions aiming to improve psychological job stressors may also benefit other health factors including mental and cardiovascular health. Future research may clarify whether such interventions can lead to a reduction of suicidal behavior, as well.

### Funding

This work was supported by AFA Insurance (an organization owned by Sweden’s labor market parties; M.Å., dnr 200265) and the Swedish state under the agreement between the Swedish government and the county councils, the ALF agreement (M.Å., ALFGBG-813511; M.W., ALFGBG-715841). The funding sources had no role in the study design, the collection, analysis and interpretation of the data, the writing of the report or the decision to submit the paper for publication.

### Conflicts of interest

The authors declare no conflicts of interest.

## Supplementary material

Supplementary material
