# Supplementary material for: Psychosocial job stressors and risk of suicidal behavior – an observational study among Swedish men
Source: Scand J Work Environ Health. 2022 Aug 31;48(6):435–45. doi: 10.5271/sjweh.4039 (PMC9888439; doi:10.5271/sjweh.4039)
Supplement: Supplementary material [file SJWEH-48-435-S001.pdf]

# Psychosocial job stressors and risk of suicidal behavior – an observational study among Swedish men[1]

*by Maria Åberg, MD, PhD,[2] Elisabeth Staats, MD, Josefina Robertson, MD, PhD, Linus Schiöler, PhD, Kjell Torén, MD, PhD, Anthony D LaMontagne, ScD, Mia Söderberg, PhD, Margda Waern, MD, PhD, Jenny Nyberg, PhD*

1. Supplementary material
2. Correspondence to: Dr Maria Åberg, School of Public Health and Community Medicine / Primary Health Care, Institute of Medicine, Box 454, SE-405 30 Gothenburg, Sweden. [E-mail: maria.aberg@gu.se]

**Table S1.** Hazard ratios (95% CIs) non-fatal suicidal behavior in working aged men, by JDC category. For each exposure category number of events during follow-up (No events) and number of events per 10 000 person-years are included.

| JDC category | Model I[a] HR (95% CI) No events/ per 10 000 person-years | Model II[b] HR (95% CI) No events/ per 10 000 person-years | Model III[c] HR (95% CI) No events/ per 10 000 person-years | Model IV[d] HR (95% CI) No events/ per 10 000 person-years |
|---|---|---|---|---|
| **Events/population** | 6420/1 476 341 | 6225/1 440 753 | 5593/1 328 058 | 5559/1 322 425 |
| **Active** | 0.50 (0.46–0.54) 1208/2.2 | 0.54 (0.49–0.58) 1171/2.1 | 0.61 (0.56–0.67) 1087/2.1 | 0.66 (0.60-0.73) 1086/2.1 |
| **High strain** | 1.11 (1.01–1.22) 943/4.5 | 1.16 (1.05–1.27) 911/4.5 | 1.17 (1.06–1.29) 833/4.4 | 1.18 (1.07-1.30) 828/4.4 |
| **Passive** | 1.60 (1.49–1.72) 3337/6.2 | 1.58 (1.47–1.70) 3247/6.2 | 1.47 (1.36–1.59) 2872/6.0 | 1.42 (1.31-1.53) 2848/6.0 |
| **Low strain** | 1 (reference) 932/4.0 | 1 (reference) 896/3.9 | 1 (reference) 801/3.8 | 1 (reference) 797/3.8 |

[a]Model I unadjusted

[b]Model II adjusted for conscription year and parental education

[c]Model III additionally adjusted for stress resilience

[d]Model IV additionally adjusted for IQ

**Table S2.** Hazard ratios (95% CIs) for non-fatal suicidal behavior in working aged men, by JDC category excluding men with a psychiatric diagnosis (F-diagnosis) at time of conscription.

| JDC category | Model I[a] HR (95% CI) No events/ per 10 000 person-years | Model II[b] HR (95% CI) No events/ per 10 000 person-years | Model III[c] HR (95% CI) No events/ per 10 000 person-years | Model IV[d] HR (95% CI) No events/ per 10 000 person-years |
|---|---|---|---|---|
| **Events/population** | 5843/1 418 703 | 5669/1 386 124 | 5115/1 279 838 | 5085/1 274 504 |
| **Active** | 0.50 (0.46–0.55) 1135/2.1 | 0.54 (0.49–0.59) 1101/2.1 | 0.61 (0.55–0.67) 1030/2.0 | 0.66 (0.60-0.73) 1029/2.0 |
| **High strain** | 1.11 (1.01–1.22) 857/4.3 | 1.15 (1.05–1.27) 829/4.2 | 1.17 (1.05–1.29) 761/4.2 | 1.17 (1.06-1.30) 756/4.2 |
| **Passive** | 1.58 (1.46–1.71) 2999/5.8 | 1.56 (1.44–1.69) 2920/5.8 | 1.45 (1.34–1.57) 2585/5.7 | 1.39 (1.28-1.51) 2564/5.7 |
| **Low strain** | 1 (reference) 852/3.8 | 1 (reference) 819/3.8 | 1 (reference) 739/3.7 | 1 (reference) 736/3.7 |

[a]Model I unadjusted

[b]Model II adjusted for conscription year and parental education

[c]Model III additionally adjusted for stress resilience

[d]Model IV additionally adjusted for IQ

**Table S3.** Hazard ratios (95% CIs) for the job strain components (job control / job demand) and risk of suicidal behavior in working aged men.

| Category | Model I[a] HR (95% CI) No events/ per 10 000 person-years | Model II[b] HR (95% CI) No events/ per 10 000 person-years | Model III[c] HR (95% CI) No events/ per 10 000 person-years | Model IV[d] HR (95% CI) No events/ per 10 000 person-years |
|---|---|---|---|---|
| **Events/population** | 8431/1 476 341 | 8162/1 440 753 | 7419/1 328 058 | 7374/1 322 425 |
| **High control** | 0.47 (0.45–0.49) 2962/3.7 | 0.50 (0.47–0.52) 2857/3.7 | 0.57 (0.54–0.59) 2649/3.6 | 0.61 (0.58-0.64) 2641/3.6 |
| **Low control** | 1 (reference) 5469/7.3 | 1 (reference) 5305/7.2 | 1 (reference) 4770/7.2 | 1 (reference) 4733/7.2 |
| **High demand** | 0.49 (0.47–0.51) 2925/3.8 | 0.53 (0.50–0.55) 2837/3.8 | 0.61 (0.58–0.64) 2645/3.7 | 0.66 (0.62-0.69) 2636/3.7 |
| **Low demand** | 1 (reference) 5506/7.1 | 1 (reference) 5325/7.1 | 1 (reference) 4774/7.0 | 1 (reference) 4738/6.9 |

[a]Model I unadjusted

[b]Model II adjusted for conscription year and parental education

[c]Model III additionally adjusted for stress resilience

[d]Model IV additionally adjusted for IQ

**Table S4.** Hazard ratios (95% CIs) for the job strain components (job control / job demand) and risk of suicidal behavior in working aged men excluding men with a psychiatric diagnosis (F-diagnosis) at time of conscription.

| Category | Model I[a] HR (95% CI) No events/ per 10 000 person-years | Model II[b] HR (95% CI) No events/ per 10 000 person-years | Model III[c] HR (95% CI) No events/ per 10 000 person-years | Model IV[d] HR (95% CI) No events/ per 10 000 person-years |
|---|---|---|---|---|
| **Events/population** | 7735/1 418 703 | 7497/1 386 124 | 6846/1 279 838 | 6806/1 274 504 |
| **High control** | 0.48 (0.46–0.50) 2771/3.6 | 0.50 (0.48–0.53) 2675/3.6 | 0.57 (0.54–0.60) 2498/3.5 | 0.61 (0.58-0.64) 2491/3.5 |
| **Low control** | 1 (reference) 4964/6.9 | 1 (reference) 4822/6.9 | 1 (reference) 4348/6.8 | 1 (reference) 4315/6.8 |
| **High demand** | 0.49 (0.47–0.52) 2728/3.7 | 0.53 (0.51–0.56) 2648/3.6 | 0.60 (0.57–0.64) 2483/3.6 | 0.65 (0.62-0.69) 2474/3.6 |
| **Low demand** | 1 (reference) 5007/6.8 | 1 (reference) 4849/6.7 | 1 (reference) 4363/6.6 | 1 (reference) 4332/6.6 |

[a]Model I unadjusted

[b]Model II adjusted for conscription year and parental education

[c]Model III additionally adjusted for stress resilience

[d]Model IV additionally adjusted for IQ
